# Regional testing of triploid hybrid clones of *populus tomentosa*

**DOI:** 10.1186/s12870-023-04304-w

**Published:** 2023-05-25

**Authors:** Liang Li, Jiahua Du, Lexun Ma, Changjun Ding, Pingdong Zhang, Xiangyang Kang

**Affiliations:** 1 State Key Laboratory of Efficient Production of Forest Tree Resources, Beijing Forestry University, Beijing, 100083 China; 2 Key Laboratory of Genetics and Breeding in Forest Trees and Ornamental Plants, Ministry of Education, Beijing Forestry University, Beijing, 100083 China; 3 College of Biological Sciences and Technology, Beijing Forestry University, Beijing, 100083 China; 4 State Key Laboratory of Tree Genetics and Breeding, Key Laboratory of Tree Breeding and Cultivation of State Forestry Administration, Research Institute of Forestry, Chinese Academy of Forestry, Beijing, 100091 China

**Keywords:** Regional test, Growth trait, Yield performance, Genetic variation, Stability analysis, Deployment zone

## Abstract

**Background:**

Triploid *Populus tomentosa*, a timber tree species, has been widely planted in northern China owing to its potential high yields and high wood quality. Though genetic variances in growth traits and wood properties have been reported across several planting sites, regional testing of triploid hybrid clones of *P. tomentosa* has not been conducted on a large scale.

**Results:**

Ten 5-year clonal trials were used to evaluate the inheritance of growth traits, to determine suitable deployment zones, and to identify optimal triploid clones at each experimental site to determine the clones that would be suitable at all sites. A total of 2,430 trees from nine triploid hybrid clones were sampled during the ten trials. The clonal and site effects and clone × site interactions were highly significant (*P* < 0.001) for all the studied growth and yield traits. The estimated repeatability of means for diameter at breast height (DBH) and tree height (H) was 0.83, which was slightly higher than for stem volume (SV) and estimated stand volume (ESV) (0.78). The Weixian (WX), Gaotang (GT), and Yanzhou (YZ) sites were each considered to be suitable deployment zones, and the Zhengzhou (ZZ), Taiyuan (TY), Pinggu (PG), and Xiangfen (XF) sites were found to be the optimal deployment zones. The TY and ZZ sites were the best discriminative environments, and the GT and XF sites were the best representative environments. GGE pilot analysis revealed that yield performance and stability were significantly different among all the studied triploid hybrid clones across the ten test sites. It was therefore necessary to develop a suitable triploid hybrid clone that could do well at each site. Taking into account both yield performance and stability, the triploid hybrid clone S2 was determined to be an ideal genotype.

**Conclusions:**

For triploid hybrid clones, the WX, GT, and YZ sites represented suitable deployment zones and the ZZ, TY, PG, and XF sites represented optimal deployment zones. Yield performance and stability were significantly different among all the studied triploid hybrid clones across the ten test sites. Developing a suitable triploid hybrid clone that could do well at all sites was therefore desirable.

## Introduction

*Populus tomentosa* Carr. (section Populus, family Salicaceae, genus *Populus*) is a tree species that is indigenous to China. As a high-quality, important, and fast-growing tree species, it is widely planted for ecological protection, landscape cultivation, and the production of timber and pulp. The first natural triploid *P. tomentosa* (2n = 3x = 57), which is thought to originate from a natural 2n pollen crossing with a normal (1n) egg, was discovered in 1993 by Zhu et al. [1]. It is characterized by its extremely large leaves and fast growth. These trees also have longer fibers and improved pulp properties [2]. The discovery and subsequent evaluations of the triploid *P. tomentosa* attracted the attention of researchers, resulting in a successful triploid breeding program using selection, hybridization, and polyploidy techniques [3–5]. As a result, a great number of triploid hybrid clones of *P. tomentosa* were produced [6]. Owing to its potentially high yields and due to the short rotation for cultivation, triploid hybrid clones of *P. tomentosa* are widely planted in northern China.

Variety regional testing, which is key to understanding new forest variety performances and market prospects, plays an important role in tree breeding [7]. Since 2000, the United States has built a regional testing network across hundreds of test sites to represent almost all types of planting environments [8]. China has also constructed a systematic regional testing network [9]. To accurately assess each variety over the long term, test sites must be highly representative of planting environments, covering several elements, including weather, soil, terrain, biological factors, etc., which are called multi-environments. Regional test results are, however, still inconsistent with actual forest results. An important reason for this is that neither the number nor the locations of the test sites can adequately represent all of the possible multi-environments.

Genotypic variation in wood properties and growth traits of triploid hybrid clones of *P. tomentosa* in three trials were reported by Zhang et al. [10]. Subsequently, genotypic parameters of basic wood density, fiber traits, and chemical properties of the nine triploid hybrid clones were estimated by Wu et al. [11] and Zhang et al. [12, 13]. Clone × site interactions were significant for the growth traits, basic wood density, fiber traits, and chemical properties, suggesting that regional testing is required before the triploid hybrid clones of *P. tomentosa* are planted on a large scale. Recently, the stability of fiber properties and growth traits in triploid hybrid clones of *P. tomentosa* were also evaluated by Wu et al. [14] according to the Shukla model [15] and the Finlay and Wilkinson stability parameter [16].

In the present study, we examined tree growth of nine triploid clones across ten trials, such as Pinggu (PG), Weixian (WX), Yanzhou (YZ), Gaotang (GT), Zhengzhou (ZZ), Changge (CG), Taiyuan (TY), Xiangfen (XF), Suzhou (SZ), and Tianmen (TM) in China. The aim of this study was threefold: (1) to evaluate the inheritance of growth traits, (2) to identify suitable deployment zones, and (3) to develop optimal triploid clones for planting. Our findings lay a foundation for developing appropriate deployment strategies for triploid hybrid clones of *P. tomentosa*.

## Results

### Variation within and among sites

The mean values, range of variation, and coefficients of phenotypic variation of all studied traits in each of the ten trials are shown in Table 1. Trees from SZ had the lowest DBH. The highest DBH was seen at the ZZ site. The difference in DBH between the lowest and highest means was 69.0% (Table 1). Trees from the TM site had the lowest H. The greatest H of trees was observed at the TY site. Among all sites, the largest SV and ESV of sampled trees were found at the ZZ site. Phenotypic variation of these composite traits (SV and ESV) at the ZZ site was, however, lower than at some of the less productive sites, such as PG, WX, TY, and TM. Joint analysis of all ten trials showed significant site effects for all studied traits (Table 2). DBH and H exhibited small phenotypic variation (coefficient of variation (CV) = 3.4–12.5%), which was much lower than the variation in SV and ESV.

**Table 1:** Mean values, ranges of variation, and coefficients of phenotypic variation (CV *p* %) of clonal means of growth and yield traits at the ten sites

Site	Traits	Mean ± SE	Range (min–max)	CV *p* %
PG	DBH (cm)	13.3 ± 1.5	8.6–14.8	11.3
	H (m)	11.6 ± 1.1	8.2–13.1	9.5
	SV (m^3^)	0.0721 ± 0.0192	0.0219–0.1011	26.5
	ESV (m^3^/ hm^2^)	118.9 ± 31.6	36.0–166.8	26.6
WX	DBH (cm)	11.1 ± 0.7	9.5–12.9	6.3
	H (m)	11.6 ± 1.2	8.3–13.4	10.3
	SV (m^3^)	0.0489 ± 0.0092	0.0219–0.1011	18.8
	ESV (m^3^/ hm^2^)	81.5 ± 15.3	51.2–116.0	18.7
YZ	DBH (cm)	10.0 ± 0.6	8.2–12.1	6.0
	H (m)	11.9 ± 0.6	10.4–13.8	5.0
	SV (m^3^)	0.0406 ± 0.0055	0.0240–0.0583	13.5
	ESV (m^3^/ hm^2^)	67.6 ± 9.2	39.9–97.1	13.6
GT	DBH (cm)	10.7 ± 0.8	8.8–11.9	7.5
	H (m)	11.8 ± 0.4	10.4–13.8	3.4
	SV (m^3^)	0.0458 ± 0.0074	0.0278–0.0616	16.2
	ESV (m^3^/ hm^2^)	76.2 ± 12.4	46.4–102.6	16.3
ZZ	DBH (cm)	14.7 ± 1.0	12.1–16.2	6.8
	H (m)	13.3 ± 1.2	10.0–15.7	9.0
	SV (m^3^)	0.0956 ± 0.0167	0.0453–0.1162	17.5
	ESV (m^3^/ hm^2^)	157.7 ± 27.6	74.7–191.7	17.5
CG	DBH (cm)	9.7 ± 0.6	8.0–11.5	6.2
	H (m)	9.4 ± 0.4	8.3–10.2	4.3
	SV (m^3^)	0.0310 ± 0.0042	0.0189–0.0464	13.5
	ESV (m^3^/ hm^2^)	51.6 ± 7.0	31.5–77.3	13.6
TY	DBH (cm)	13.6 ± 1.7	6.8–16.4	12.5
	H (m)	13.9 ± 1.4	9.8–16.0	10.1
	SV (m^3^)	0.0867 ± 0.0257	0.0160–0.0464	29.6
	ESV (m^3^/ hm^2^)	144.6 ± 42.3	26.4–207.9	29.3
XF	DBH (cm)	11.6 ± 0.5	10.5–13.3	4.3
	H (m)	13.1 ± 0.5	11.9–1.2	3.8
	SV (m^3^)	0.0581 ± 0.0067	0.0439–0.0786	11.5
	ESV (m^3^/ hm^2^)	96.8 ± 11.2	73.1–131.0	11.6
SZ	DBH (cm)	8.7 ± 0.3	7.3–9.7	3.4
	H (m)	9.4 ± 0.5	8.6–10.9	5.3
	SV (m^3^)	0.0250 ± 0.0026	0.0439–0.0786	10.4
	ESV (m^3^/ hm^2^)	41.5 ± 4.4	26.1–54.9	10.6
TM	DBH (cm)	9.0 ± 1.1	5.2–11.6	12.2
	H (m)	8.8 ± 0.7	5.6–10.6	8.0
	SV (m^3^)	0.0278 ± 0.0096	0.0439–0.0786	34.5
	ESV (m^3^/ hm^2^)	45.9 ± 15.8	9.6–105.6	34.4

**Table 2:** Summary of results of analysis of variance and estimated repeatability for tree growth and yield traits at the ten sites combined

Trait	*P* value			*R*_c_^2^	Percent
	Clones	Sites	Sites × clones		
DBH	0.000	0.000	0.000	0.83 ± 0.07	43.8
H	0.000	0.000	0.000	0.83 ± 0.07	43.4
SV	0.000	0.000	0.000	0.78 ± 0.09	32.6
ESV	0.000	0.000	0.000	0.78 ± 0.09	32.3

### Clonal variation and repeatability

The results of the analysis of variance for the ten sites combined are presented in Table 2. Significant differences in both growth and yield traits were found among the clones (Table 2). For all studied traits, variance due to error accounted for most of the variation in these traits, varying from 39.3 to 51.8% of the total variation. Most of the variance in DBH and H (21.6%) was, however, due to the clone. DBH and H in the triploid hybrid clones of *P. tomentosa* had the highest estimated repeatability (0.83).

The estimated repeatability for the mean values for tree growth and yield traits at individual sites is shown in Table 3. In the present study, we observed no significant differences in DBH, H, SV, and ESV among the triploid clones at the YZ and TM sites. Consequently, we did not estimate the repeatability for these four traits. Estimated clonal repeatability for DBH varied from 0.60 to 0.94 and from 0.62 to 0.94 for H. Estimated clonal repeatability of SV and ESV ranged from 0.76 to 0.94.

**Table 3:** Results of analysis of variance and estimated repeatability for tree growth and yield traits at each site

Site		Trait			
		DBH	H	SV	ESV
PG	*P* value	0.975	0.000	0.000	0.000
	*R*_*c*_^2^	-	0.93	0.94	0.94
WX	*P* value	0.027	0.000	0.002	0.002
	*R*_*c*_^2^	0.66	0.93	0.81	0.81
YZ	*P* value	0.076	0.072	0.060	0.060
	*R*_*c*_^2^	-	-	-	-
GT	*P* value	0.000	0.997	0.002	0.002
	*R*_*c*_^2^	0.94	-	0.81	0.81
ZZ	*P* value	0.000	0.000	0.000	0.000
	*R*_*c*_^2^	0.88	0.94	0.88	0.88
CG	*P* value	0.049	0.011	0.076	0.076
	*R*_*c*_^2^	0.60	0.73	-	-
TY	*P* value	0.010	0.002	0.002	0.002
	*R*_*c*_^2^	0.73	0.80	0.80	0.80
XF	*P* value	0.005	0.011	0.001	0.001
	*R*_*c*_^2^	0.77	0.73	0.83	0.83
SZ	*P* value	0.357	0.043	0.285	0.285
	*R*_*c*_^2^	-	0.62	-	-
TM	*P* value	0.122	0.251	0.100	0.100
	*R*_*c*_^2^	-	-	-	-

### Determination of deployment zone

**Fig. 1:**
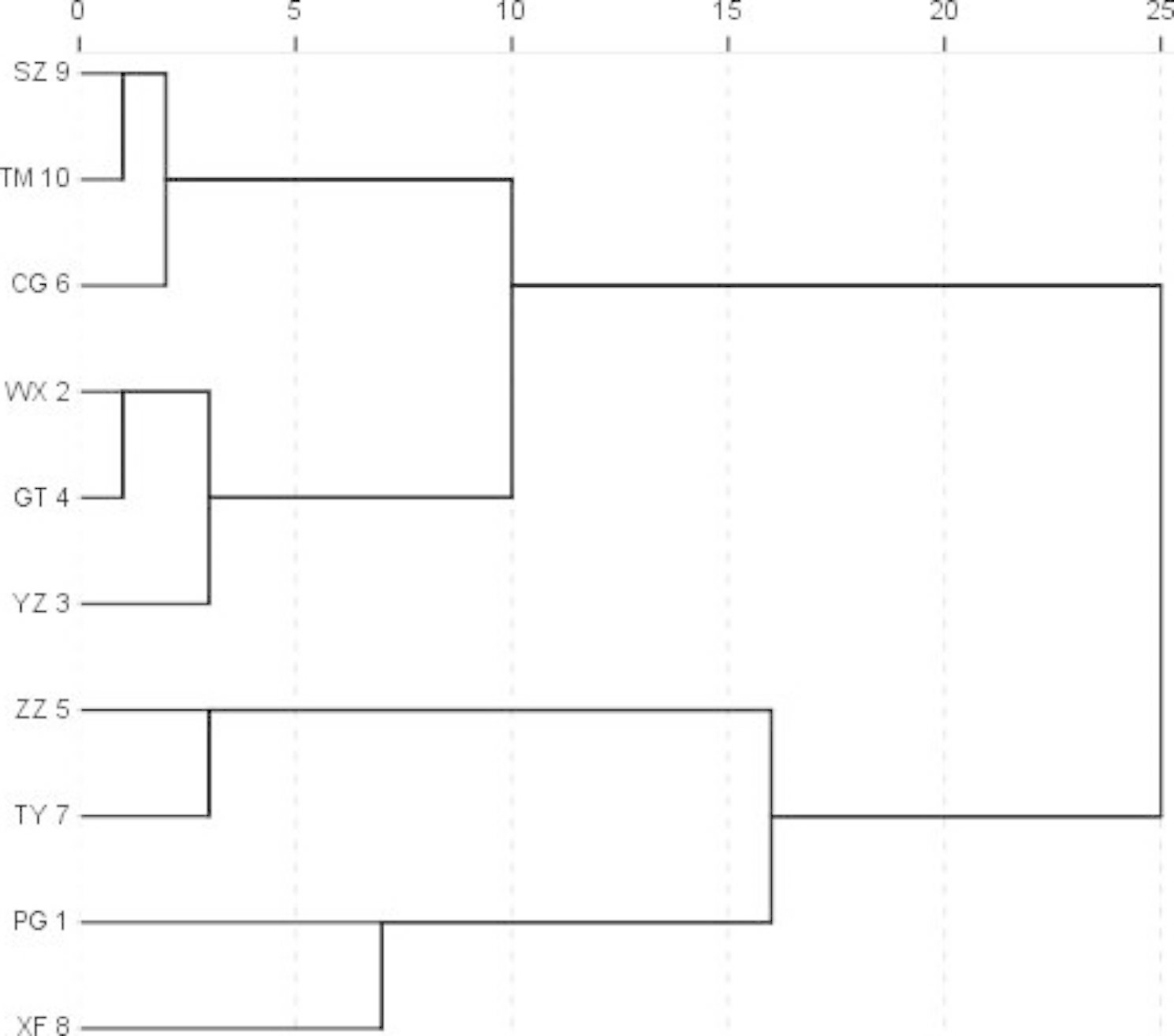
Cluster analysis of yield in triploid hybrid clones of *P. tomentosa* across the ten trials

Cluster analysis of ESV of triploid hybrid clones of *P. tomentosa* across the ten trials was conducted using Euclidean Distance (Fig. 1). The ten trials were classified into four subgroups. The three least productive sites (SZ, TM, and CG) were regarded as non-suitable deployment zones. The intermediately productive sites (WX, GT, and YZ) were considered to be suitable deployment zones, and the four most highly productive sites (ZZ, TY, PG, and XF) sites were considered to be optimal deployment zones.

### GGE analysis of growth and yield traits

**Fig. 2:**
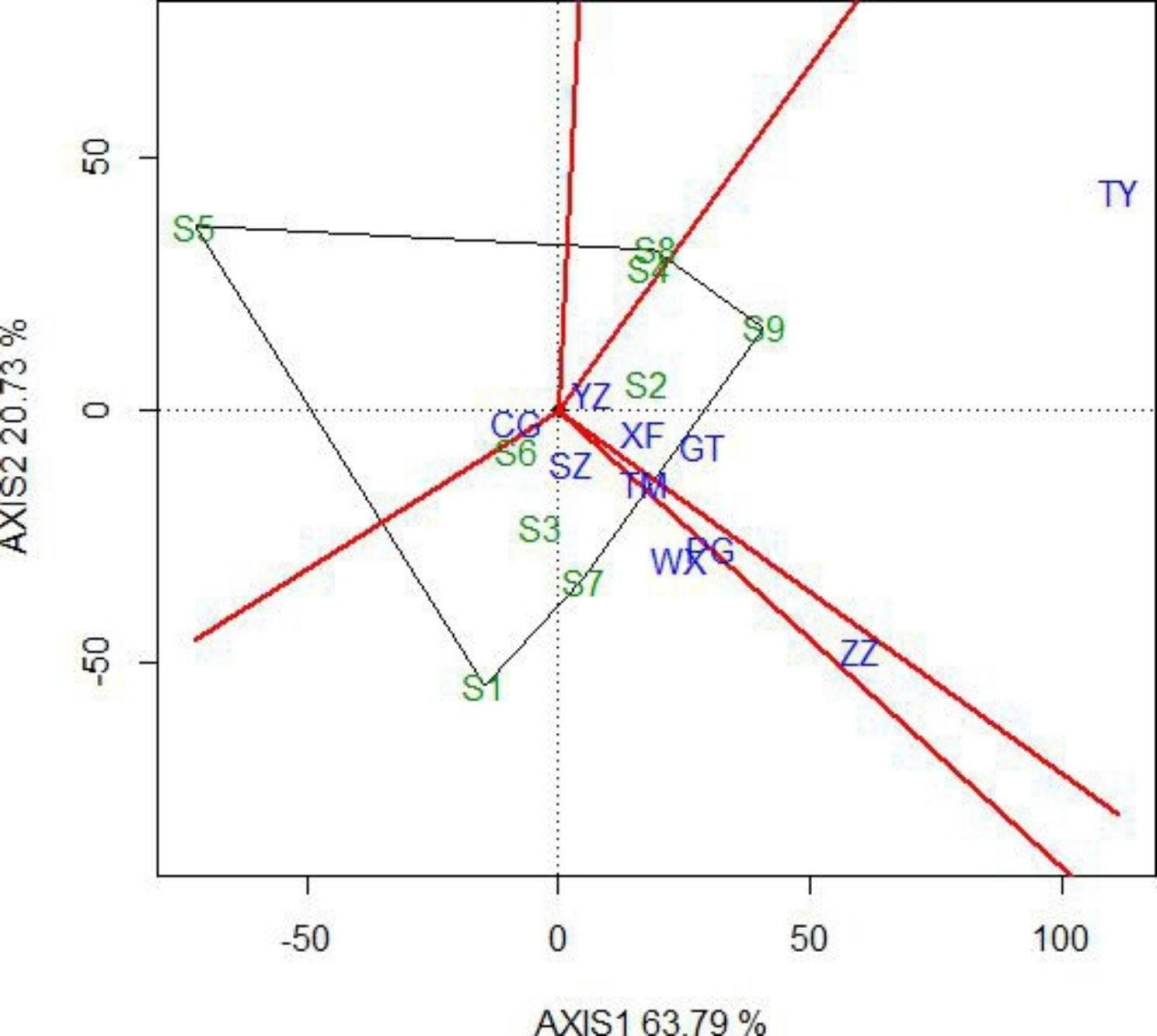
The Which-Won-Where view of the GGE biplot of triploid hybrid clones of *P. tomentosa*. The green numbers represent triploid hybrid clones and the blue characters represent sites

The ‘‘Which-Won-Where’’ function of the GGE biplot presents the outermost genotypes as a polygon and makes a vertical line for each edge of the polygon through the origin. Based on the graphical results, the test environments were categorized, with the superior triploid clones identified in each group. The results showed that the ten test sites could be divided into four groups: the TY, YZ, XF, and GT sites in one group; the TM, PG, and ZZ sites in the second group; the SZ and WX sites in the third group; and the CG site in an independent group (Fig. 2). The triploid hybrid clone S9 was the highest productive genotype at the TY, YZ, XF, and GT sites. Clone S7 was the highest productive genotype at the TM, PG, and ZZ sites, and clone S1 was the highest productive genotype at the SZ and WX sites.

**Fig. 3:**
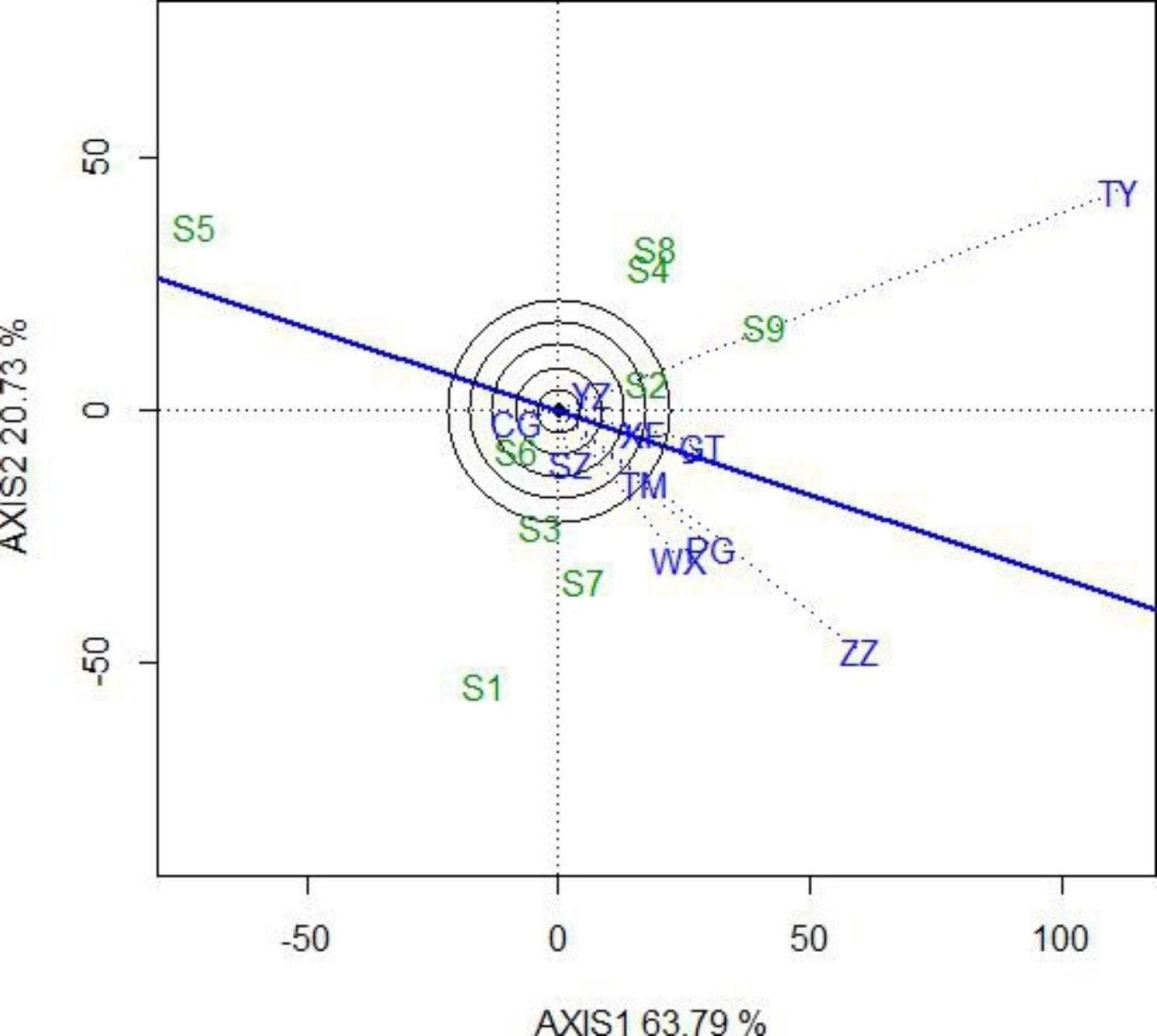
Discrimination and representativeness of the GGE biplot of triploid hybrid clones of *P. tomentosa*. The green numbers represent triploid hybrid clones and the blue characters represent sites

The choice of the test environment is in direct relation to the reliability of variety breeding. An ideal test environment should be strongly discriminative to test varieties and be representative of mega-environments. Figure 3 shows the discrimination and representativeness of the test sites. The blue line with arrows shows the average environment axis and sites with long vectors in relation to the origin represent high discrimination. The angle between the test environment vector and the average environment axis represents the representativeness of the test environment. The smaller the angle, the stronger the representativeness of the test environment. The results show that the TY and ZZ sites were the best discriminative environments, and the GT and XF sites were the best representative environments (Fig. 3). The CG site was considered as an unsuitable test environment.

### Genotype-environment interaction and stability analysis of yield

In the present study, a significant interaction of clone × site was found for all studied growth and yield traits (Table 2). Compared to other studied traits, ESV had a higher ratio of clone × site interaction variance compared to the sum of clonal and clone × site interaction variances (67.7%).

**Fig. 4:**
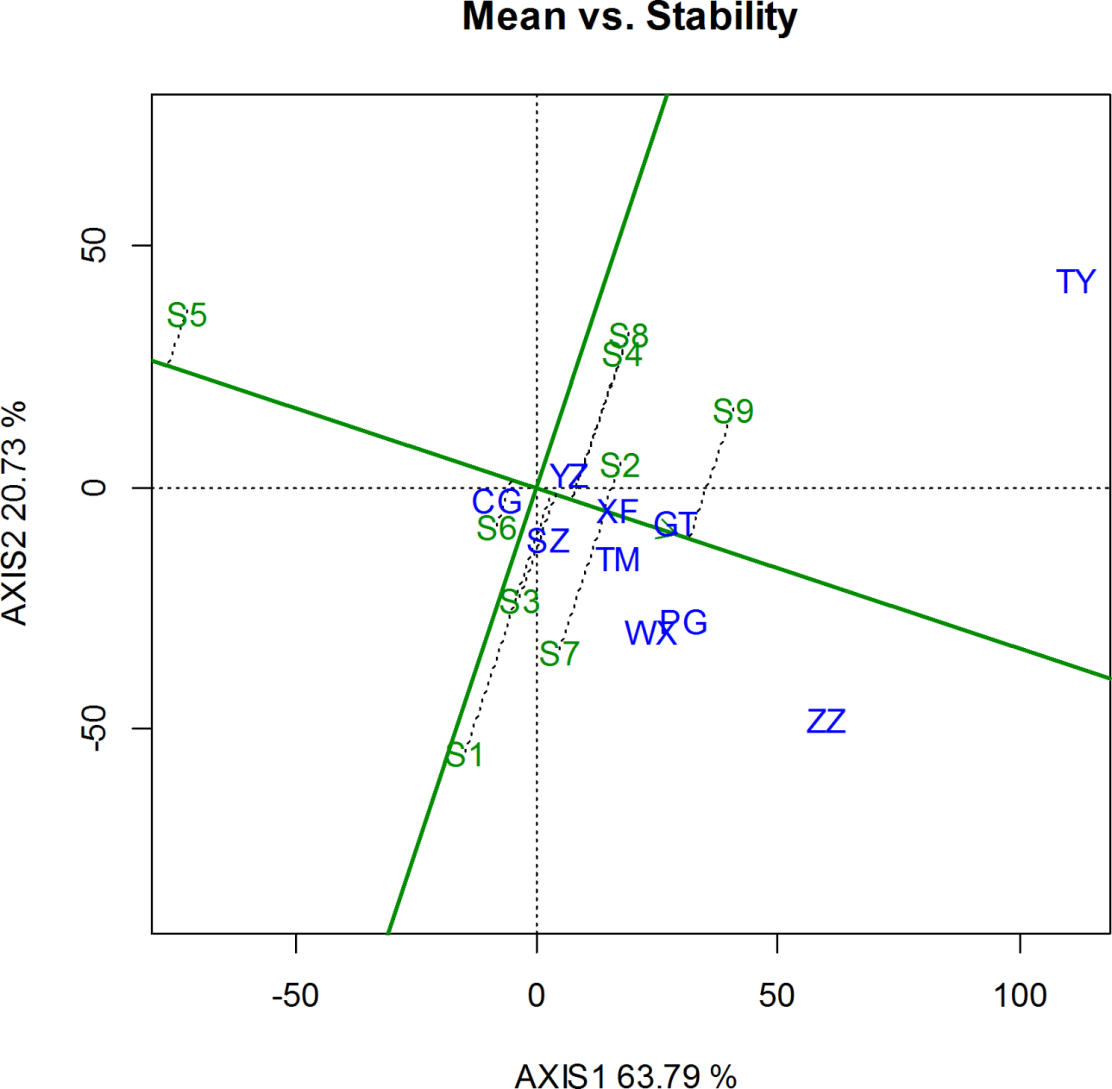
Yield performance and stability analysis of the GGE biplot of triploid hybrid clones of *P. tomentosa*. The green numbers represent triploid hybrid clones and the blue characters represent sites

The high yield and stability of the tested clones are shown in Fig. 4. The vertical black dotted line represents the average yield and stability of each triploid hybrid clone across all environments. The coordinate values on the average environmental axis reflect the yield of the varieties, and the longer dotted line shows that the yield was more unstable.

The solid green straight lines through the origin represent the grand (overall) means. The genotype on the left side of the green more-vertical line indicates a yield below the grand mean, while the genotype on the right side has a yield above the grand mean. Yield performance and stability were noticeably different among all studied triploid hybrid clones across the ten test sites. The triploid hybrid clone S5 had the lowest yield, and clones S6, S1, S3, S4, and S8 were around the overall mean, while clone S9 had the highest yield. The most stable triploid hybrid clones were S2, S6, and S5, and the most unstable were clones S1, S8, S7, S9, and S4. Taking into account both high yield and stability, triploid hybrid clone S2 was, therefore, the most ideal genotype.

## Discussion

### Variations among sites

**Fig. 5:**
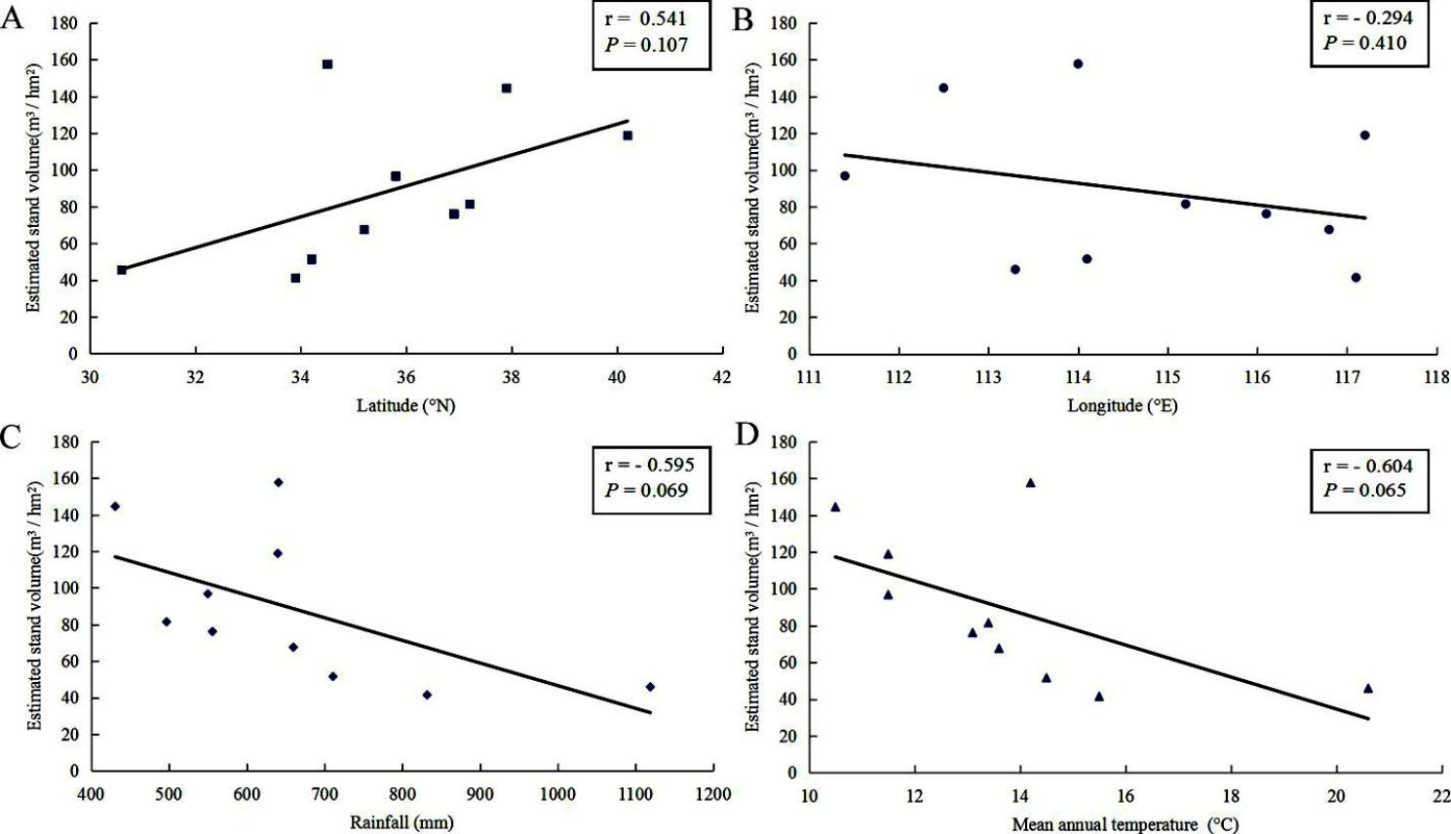
Correlations between estimated stand volume and (**A**) latitude, (**B**) longitude, (**C**) rainfall, and (**D**) mean annual temperature

Site effects reflect the response of trees to the combined effects of edaphic as well as local and regional climatic conditions [14, 17]. Even if the clonal trials in this study were not designed to separate these various effects, some conclusions could still be drawn. The poor field performance for growth at SZ was probably due to poor drainage (because of soil compaction) and the wet weather during the test period of the trial. A slower growth rate at TM was also observed because TM is out of the traditional distribution zone of *P. tomentosa*. The joint analysis of all ten trials exhibited significant site effects for all the studied growth traits (Table 2). Additionally, a positive correlation between ESV and latitude and negative correlations between ESV and longitude as well as rainfall and mean annual temperature were seen (Fig. 5), indicating that edaphic and regional climatic conditions did have significant influences on tree growth. At all the ten sites, the DBH and H of the nine triploid clones had less phenotypic plasticity than SV and ESV, as evidenced by the lower CV values, indicating that the site impacts DBH and H less than SV and ESV.

### Clonal variation and repeatability

Clonal effects in the joint analysis for all studied growth and yield traits were significant (*P* < 0.001). Higher significance of clonal effects was seen at the ZZ, XF, and TY sites (Table 3), suggesting that these three sites resulted in much larger genetic variations and small environment variations than the other less productive sites. A significant clonal effect in the growth traits of poplars or their triploid hybrids has also been documented in previous studies [14, 18–21].

Precise estimation of quantitative parameters requires, for the purpose of tree improvement, more testing sites for the same genotypes. An increase in the number of clonal testing locations would contribute to improving the accuracy of clonal effects, resulting in a reduction in the complexity of field testing. A previous study [10] reported that the estimated repeatability of DBH, H, and SV in the same nine triploid clones of *P. tomentosa* at three testing sites varied from 0.55 to 0.62. When the number of testing sites was six, the estimated repeatability of DBH, H, and SV changed from 0.76 to 0.85 [14]. In the present study, we estimated the repeatability of growth and yield traits of nine triploid clones across ten testing sites. The estimated repeatability of DBH, H, SV, and ESV were 0.83, 0.83, 0.78, and 0.78, respectively. Compared with the estimated repeatabilities of growth and yield traits across the six testing sites, no significant differences in the estimated repeatability of growth and yield traits were found, suggesting that six testing sites are sufficient to estimate quantitative parameters in triploid clones of *P. tomentosa*.

### Determination of deployment zone

Different tree species have different distribution zones. For example, wild types of *P. tomentosa* can be found in the Beijing, Hebei, Henan, Shandong, Shanxi, Shaanxi, Gansu, Jiangsu and Anhui provinces, and the Ningxia autonomous region [1]. The distribution zone of *P. tomentosa* is about 1 million square kilometers, which represents one-tenth of China’s land area. In this study, the ten testing sites of triploid hybrid clones of *P. tomentosa* were divided into three deployment zones: non-suitable deployment zone, suitable deployment zone, and optimal deployment zone. The SZ, TM, and CG sites were identified as non-suitable deployment zones; the WX, GT and YZ sites as suitable deployment zones; and the ZZ, TY, PG, and XF sites as optimal deployment zones. This information is valuable for guiding the pulp and paper industry as to where to plant the triploid hybrid clones of *P. tomentosa* to maximize yield. Compared with wild types of *P. tomentosa*, however, the size of the suitable deployment and optimal deployment zones was much smaller. This may be partially explained by the differences in distribution zones for the female parents of triploid hybrid clones.

### Clone × site interaction and stability

As regional testing approaches can aid the development of clones suitable for different environments, clone × site interaction might have a significant effect on the accuracy of breeding values, thus resulting in a decrease in genetic gain. With results differing among testing sites and deployment zones, clone × site interaction could lead to bias in the estimates and thus a reduction in genetic gain if improperly accounted for [22]. Significant clone × site interactions for DBH, H, and SV were seen (Table 2). This finding agrees with former studies on the presence of significant clone × site interaction for growth traits of poplar hybrid clones or triploid hybrid clones [13, 14, 18]. For yield traits (ESV), clone × site interaction was also significant in the present study, which might be sufficient to justify detailed clonal testing in order to obtain optimal clonal deployment.

For the poplar breeding program, it is critical to estimate the yield of a genotype in different locations. By doing so, the stability of a genotype may be uncovered via regression coefficients [16] and stability variances [15]. Wu et al. [14] reported that the higher regression coefficient means of the triploid clones B304, B330 and B331 for SV suggest that these clones have higher norms of reaction to the improvement in testing sites and higher phenotypic plasticity. Zhang et al. [21] evaluated the interactions between environment and growth traits and the stability of new triploid clones of *P. tomentosa* in five trials, and an ideal triploid clone B303 was screened. The stability of tree varieties may also be evaluated through additive main effects and multiplicative interaction (AMMI) and the GGE biplot [23–25]. In this study, we evaluated the stability of triploid hybrid clones of *P. tomentosa* using GGE biplot analysis. The yield performance and stability were noticeably different among all the studied triploid hybrid clones across all testing sites. This calls for the selection of clones separately for each site. The ‘‘Which-Won-Where’’ function of the GGE biplot revealed that the triploid hybrid clone S9 was the highest productive genotype at the TY, YZ, XF and GT sites. Clone S7 was the highest productive genotype for the TM, PG and ZZ sites, and clone S1 was the highest productive genotype for the SZ and WX sites.

## Conclusions

Based on the regional testing and analyses of triploid hybrid clones of *P. tomentosa* across the ten testing sites, we concluded that the clonal and site effects and clone × site interactions had a highly significant effect (*P* < 0.001) on all the studied growth and yield traits. The estimated repeatability of means for diameter at breast height (DBH) and tree height (H) was 0.83, which was slightly higher than for stem volume (SV) and estimated stand volume (ESV) (0.78). The WX, GT, and YZ sites were suitable deployment zones, and the ZZ, TY, PG, and XF sites represented optimal deployment zones. The TY and ZZ sites were the best discriminative environments and the GT and XF sites were the best representative environments. The GGE pilot analysis revealed that yield performance and stability were noticeably different among all the studied triploid hybrid clones across ten test sites. Developing a suitable triploid hybrid clone for each site is, therefore, necessary. Taking into account both yield performance and stability, the triploid hybrid clone S2 was determined to be an ideal genotype.

## Methods

### Experimental design

**Fig. 6:**
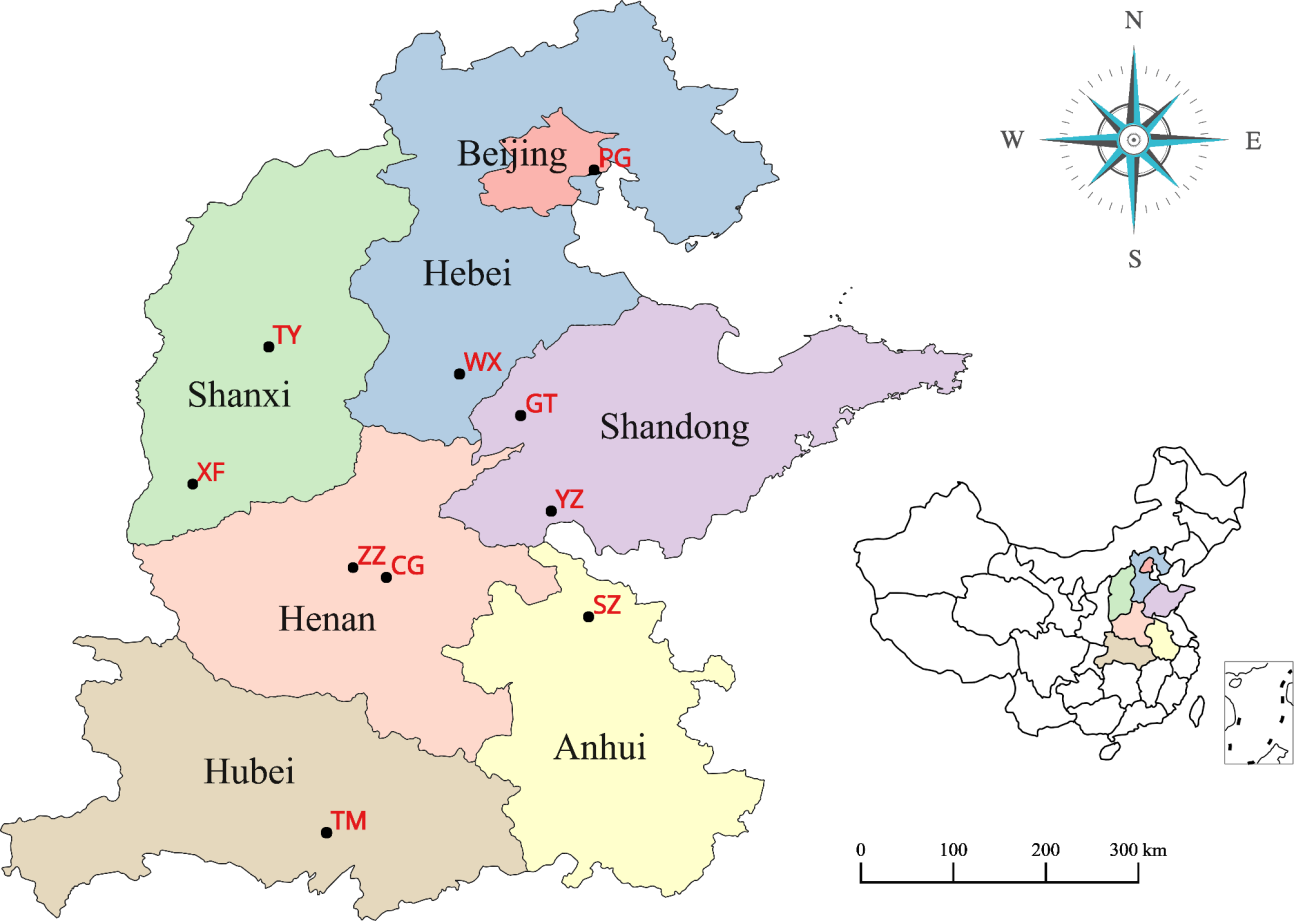
Geographical locations of the ten clonal trials in this study

In this study, the materials used were sampled from ten triploid hybrid poplar trials conducted in China. These trials were designed and established by the triploid breeding program of Beijing Forestry University using cuttings on sandy loam with typical soil fertility. The clonal trials at WX, YZ, GT, SZ, CG, and XF were established in the spring of 2004 and the clonal trials at PG, ZZ, TY, and TM were set up in the spring of 2005. The characteristics of the ten trials are presented in Table 4. The specific geographic locations of the ten clonal trials are shown in Fig. 6. The planted cuttings were approximately 20–25 cm in length. Each trial was repeated three times. Nine triploid hybrid clones were involved in each clonal trial (Table 5). The nine triploid hybrid clones were pre-selected among the triploids derived from five parent trees with fast growth [1]. A randomized complete block design with three replicates was used in each trial (240 trees per plot, resulting in a total of 720 trees per clone per trial). Nine poplar clones were planted on rectangular plots at each site, and each plot included 240 trees (4 × 60 trees) with 2 m × 3 m spacing. No thinning was conducted during the testing period.

**Table 4:** Location, climatic conditions and description of ten trials

Site	Latitude (°N)	Longitude (°E)	Mean annual Temperature (°C)	Rainfall (mm/year)	Number of clones	Sample trees
PG	40°13’	117°12’	11.5	640	9	270
WX	37°12’	115°14’	13.4	497	9	270
YZ	35°10’	116°49’	13.6	660	9	270
GT	36°51’	116°04’	13.1	556	9	270
ZZ	34°27’	113°57’	14.2	641	9	270
CG	34°12’	114°06’	14.5	711	9	270
TY	37°52’	112°33’	10.5	431	9	270
XF	35°50’	111°21’	11.5	550	9	270
SZ	33°54’	117°04’	15.5	832	9	270
TM	30°36’	113°18’	20.6	1119	9	270

**Table 5:** Identity and origin of the hybrid clones

No.	Clone identity	Parents	Sex
1	S1	(*P. tomentosa* × *P. bolleana*) × *P. tomentosa*	female
2	S2	(*P. tomentosa* × *P. bolleana*) × *P. tomentosa*	female
3	S3	(*P. tomentosa* × *P. bolleana*) × *P. tomentosa*	female
4	S4	(*P. tomentosa* × *P. bolleana*) × *P. tomentosa*	female
5	S5	(*P. alba* × *P. glandulosa*) × *P. tomentosa*	female
6	S6	(*P. alba* × *P. glandulosa*) × *P. tomentosa*	female
7	S7	(*P. tomentosa* × *P. bolleana*) × *P. tomentosa*	female
8	S8	(*P. tomentosa* × *P. bolleana*) × *P. tomentosa*	male
9	S9	(*P. tomentosa* × *P. bolleana*) × *P. tomentosa*	female

### Measurement of growth and yield traits

In each trial, ten trees were randomly sampled from each plot per clone, resulting in a total of 270 randomly selected trees per location. 2,700 sampled trees were therefore collected in total for the ten clonal trials. After 5 years of growth, the DBH and H of all sampled trees were measured. The SV of each sample was estimated according to the volume function of DBH and H as described by Chen et al. [26].

The ESV was calculated using the following equation (Eq. 1):

1 $${\rm{ESV}}\,{\rm{ = }}\,{\rm{SV}}\,{\rm{ \times }}\,{\rm{N}}$$

where ESV is the estimated stand wood volume in m^3^/ hm^2^, SV is the mean stem volume of each plot in each replicate in m^3^, and N is the number of survival trees per square kilometer.

### Statistical analysis

Analysis of variance of DBH, H, SV, and ESV was performed using the UNIVARIATE procedure of the SPSS software (SPSS for Windows, version 18.0, SPSS, Chicago, IL). Variation among ramets of the sampled clones was analyzed using analysis of variance, using a linear model (Eq. 2) within the site:

2 $${X_{ik}}_ = \mu + {C_i} + {\varepsilon _{ik}}$$

where *X*_*ik*_ is an observation on the *k*th ramet from the *i*th clone, *µ* is the general mean, *C*_*i*_ is the effect due to the *i*th clone, and *ε*_*ik*_ is random error.

Repeatability of means within a site was calculated using the following formula (3):

3 $$R_c^2 = \frac{{\hat \sigma _c^2}}{{\hat \sigma _c^2 + \frac{{\hat \sigma _e^2}}{k}}}$$

The linear model (4) was used for joint analyses of the ten sites together [18]:

4 $${X_{ijk}} = \mu + {C_i} + {\rm{ }}{L_j} + {\rm{ }}{C_i}{L_j} + {\varepsilon _{ijk}}$$

where *X*_*ijk*_ is an observation on the *k*th ramet from the *i*th clone in the *j*th location; *µ* is the overall mean; *C*_*i*_ is the effect due to the *i*th clone; *L*_*j*_ is the effect due to the jth location; *C*_*i*_*L*_*j*_ is the interaction between the *i*th clone and *j*th location; and *ε*_*ijk*_ is random error. All terms were considered random, except for location which was considered a fixed effect.

The repeatability of clone means was calculated according to the following formula (5):

5 $$R_c^2 = \frac{{\hat \sigma _c^2}}{{\frac{{k2\hat \sigma _c^2}}{{k2}} + \frac{{k1\hat \sigma _{LXC}^2}}{{k2}} + \frac{{\hat \sigma _e^2}}{{k2}}}}$$

where *k*_*1*_ is the coefficient associated with the variance due to the clone × location interaction term (*σ*^*2*^_*L×C*_), and *k*_*2*_ is the coefficient associated with the variance due to clonal variation (*σ*^*2*^_*C*_). Approximate standard errors (SE) for repeatability estimates were calculated based on the following formula [27]:

6 $${\rm{SE}}({{\rm{R}}_{\rm{C}}}^{\rm{2}}){\rm{ = }}\sqrt {\frac{{{\rm{2}}{{({\rm{1 - }}R_C^2)}^{\rm{2}}}{{[{\rm{1 + }}({k_2}{\rm{ - 1}})R_C^2]}^{\rm{2}}}}}{{{k_2}({k_2}{\rm{ - 1}})(N - {\rm{1}})}}}$$

where N is the number of clones tested.

The GGE model equation for the first two principal components [28] is written as.

7 $${y_{ij}} - {\beta _j} = {\lambda _1}{\xi _i}_1{\eta _j}_1 + {\lambda _2}{\xi _i}_2{\eta _j}_2 + {\varepsilon _{ij}}$$

where *y*_*ij*_ is the measured mean for the *i*_th_ clone at the jth site, *β*_*j*_ is the measured mean for all clones at the *j*_th_ site, *λ*_1_ and *λ*_2_ are the singular values for the first two principal components (PC1 and PC2), *ξ*_*i*1_ and *ξ*_*i*2_ are the scores of clone *i* for PC1 and PC2, *η*_*j*1_ and *η*_*j*2_ are the scores for site *j* for PC1 and PC2, and *ε*_*ij*_ is the residual error.

GGE biplot analysis was employed using the R package GGEBiplotGUI [29]. GGE biplots were based on singular value decomposition with symmetrical scaling and focused on the environment [30]. Cluster analysis of yields for the ten sites was performed using the CLASSIFICATION procedure of the SPSS software.

## Data Availability

All data generated or analyzed during this study are included in this published article.

## Abbreviations

CV *p* %: Coefficients of phenotypic variation
CV: Coefficient of variation
H: Tree height
DBH: Diameter at breast height
SV: Stem volume
ESV: Estimated stand volume
*R*_c_^2^: Repeatability of clonal means
GGE: Genotype main effect plus genotype × environment interaction
AMMI: Additive main effects and multiplicative interaction
SE: Standard errors
SZ: Suzhou
TM: Tianmen
CG: Changge
WX: Weixian
GT: Gaotang
YZ: Yanzhou
ZZ: Zhengzhou
TY: Taiyuan
PG: Pinggu
XF: Xiangfen
*P*: Probability levels
